# Study and characterization of mutated antigen specific T cells isolated from fresh tumor and peripheral lymphocytes in cancer patients

**DOI:** 10.1186/2051-1426-3-S2-P7

**Published:** 2015-11-04

**Authors:** Cyrille Cohen, Jared J Gartner, Miryam Horovitz-Fried, Katerina Shamalov, Kasia Trebska-Mcgowan, Valery Bliskovsky, Maria R Parkhurst, Chen Ankri, Todd D Prickett, Jessica Crystal, Yong Li, Mona El-Gamil, Steven A Rosenberg, Paul F Robbins

**Affiliations:** 1 Bar-Ilan University, Ramat Gan, Israel; 2 Surgery Branch/National Cancer Institute / National Institutes of Health, Bethesda, MD, USA; 3 NIH/National Cancer Institute, Bethesda, MD, USA; 4 NIH, Bethesda, MD, USA; 5 NCI/NIH, Bethesda, MD, USA; 6 NIH/NCI, Bethesda, MD, USA

## Background

T cell-based immunotherapy shows promise for the successful treatment of advanced cancer. Indeed, adoptively transferred tumor infiltrating T lymphocytes (TIL) that mediated complete regression of metastatic melanoma have been shown to recognize neoantigens/mutated epitopes expressed by autologous tumors.

## Methods

In the present study, we sought to develop a strategy for facilitating the isolation, expansion and study of T cells specific for neoantigens. We performed whole exome sequencing on matched tumor and normal DNA from eight metastatic melanoma patients. Candidate neo-epitopes, identified using a peptide/MHC binding algorithm, were synthesized and we used those to produce panels of MHC/tetramers that were evaluated for binding to tumor digests and cultured TIL used for patient treatment.

## Results

This resulted in the characterization of nine mutated epitopes from five of eight patients tested. Cells reactive with eight of the nine epitopes could be isolated from autologous peripheral blood where they were detected at frequencies that were estimated to range between 0.4% and 0.002%.

## Conclusions

To the best of our knowledge, this represents the first demonstration of the successful isolation of mutation reactive T cells from patient peripheral blood prior to immune therapy. Moreover, we were able to rapidly isolate and clone from these cells TCRs specific for neoantigens that could be used to endow T cells with mutated antigens specificity. In addition, neo-antigens reactive T cells were detected in the patient peripheral blood for up to one year after treatment. We believe this potentially provides the basis for designing novel personalized immunotherapies for treating patients with advanced cancer.

